# TSC22D4 is a molecular output of hepatic wasting metabolism

**DOI:** 10.1002/emmm.201201869

**Published:** 2013-01-11

**Authors:** Allan Jones, Kilian Friedrich, Maria Rohm, Michaela Schäfer, Carolyn Algire, Philipp Kulozik, Oksana Seibert, Karin Müller-Decker, Tjeerd Sijmonsma, Daniela Strzoda, Carsten Sticht, Norbert Gretz, Geesje M Dallinga-Thie, Barbara Leuchs, Manfred Kögl, Wolfgang Stremmel, Mauricio Berriel Diaz, Stephan Herzig

**Affiliations:** 1 Joint Division Molecular Metabolic Control, DKFZ-ZMBH Alliance, Network Aging Research, German Cancer Research Center (DKFZ) Heidelberg, Center for Molecular Biology (ZMBH) and University Hospital, Heidelberg UniversityHeidelberg, Germany; 2 Dept. of Gastroenterology, University Hospital HeidelbergHeidelberg, Germany; 3 Core Facility Tumor Models, DKFZHeidelberg, Germany; 4 Medical Research Center, Klinikum MannheimMannheim, Germany; 5 Dept. of Vascular Medicine, AMC AmsterdamAmsterdam, Netherlands; 6 Division of Tumor Virology, DKFZHeidelberg, Germany; 7 Genomics and Proteomics Core Facility, DKFZHeidelberg, Germany

**Keywords:** cancer cachexia, lipid metabolism, liver, transcription, TSC22D4

## Abstract

In mammals, proper storage and distribution of lipids in and between tissues is essential for the maintenance of energy homeostasis. Here, we show that tumour growth triggers hepatic metabolic dysfunction as part of the cancer cachectic phenotype, particularly by reduced hepatic very-low-density-lipoprotein (VLDL) secretion and hypobetalipoproteinemia. As a molecular cachexia output pathway, hepatic levels of the transcription factor transforming growth factor beta 1-stimulated clone (TSC) 22 D4 were increased in cancer cachexia. Mimicking high cachectic levels of TSC22D4 in healthy livers led to the inhibition of hepatic VLDL release and lipogenic genes, and diminished systemic VLDL levels under both normal and high fat dietary conditions. Liver-specific ablation of TSC22D4 triggered hypertriglyceridemia through the induction of hepatic VLDL secretion. Furthermore, hepatic TSC22D4 expression levels were correlated with the degree of body weight loss and VLDL hypo-secretion in cancer cachexia, and TSC22D4 deficiency rescued tumour cell-induced metabolic dysfunction in hepatocytes. Therefore, hepatic TSC22D4 activity may represent a molecular rationale for peripheral energy deprivation in subjects with metabolic wasting diseases, including cancer cachexia.

## INTRODUCTION

Cancer-induced cachexia describes a multi-factorial disease condition characterized by massive loss of adipose tissue and skeletal muscle mass and is believed to be responsible for up to 30% of cancer-related deaths in humans (Fearon et al, 2012). Due to the phenotypic heterogeneity of cancer cachexia, which is dependent on tumour type, size and mass (Dewys et al, 1980), and the mostly unknown etiology at the molecular level, cachexia still represents an immediate unmet medical need as effective and routine therapeutic measures are still lacking to date (Tisdale, 2002). Whereas cancer cachexia has traditionally been seen as a tumour-initiated event that eventually deprives host energy resources to feed tumour growth (Theologides, 1979), more recent studies highlight the role of cachectic metabolism as an integrative, global response to noxious stimuli, including tumours but also septic or traumatic conditions (Fearon et al, 2012). Indeed, the clinical severity of cancer cachexia hardly correlates with tumour mass (Martignoni et al, 2005), indicating that the tumour controls peripheral energy balance in critical host tissues, rather than acting as a direct ‘energy sink’.

The vast majority of previous studies have focused on the loss of skeletal muscle and adipose tissue as phenotypic features of cancer cachexia. However, the frequently documented steatohepatitis in experimental and human cancer cachexia and its impact on overall energy homeostasis suggests that the liver also plays an important but as-yet underestimated role in the metabolic performance during cancer cachexia (Martignoni et al, 2009; Teli et al, 1995; Tisdale, 2002). In this respect, hepatic metabolic pathway activities can significantly contribute to overall energy balance during tumour cachexia as classically exemplified by the lactate-to-glucose Cori cycle between tumours and the liver (Holroyde et al, 1975). This suggests that alterations in hepatic glucose and lipid homeostasis may provide critical cues for the manifestation of cancer cachexia but remain largely undefined.

Recent studies in mice and men have identified a number of transcriptional regulators as critical checkpoints in dysfunctional liver energy homeostasis during opposing states of excessive energy availability, i.e. obesity, as exemplified by nuclear receptor co-factors peroxisome proliferator-activated receptor co-activator (PGC) 1, receptor interacting protein (RIP) 140, the steroid hormone receptor co-activators (SRC), and transducin beta-like (TBL) 1 (Berriel Diaz et al, 2008; Feige & Auwerx, 2007; Herzig et al, 2001; Kulozik et al, 2011). Given the importance of transcription factors and their co-regulators as downstream integrators of cachectic responses in other metabolic tissues, including skeletal muscle (Sandri et al, 2004; Zhao et al, 2007; Zhou et al, 2010), we tested the hypothesis that transcriptional co-regulators represent critical molecular output pathways of hepatic energy handling under tumour-bearing, cachectic conditions.

## RESULTS

### Cancer cachexia disturbs hepatic TG handling and export

**Figure 1:**
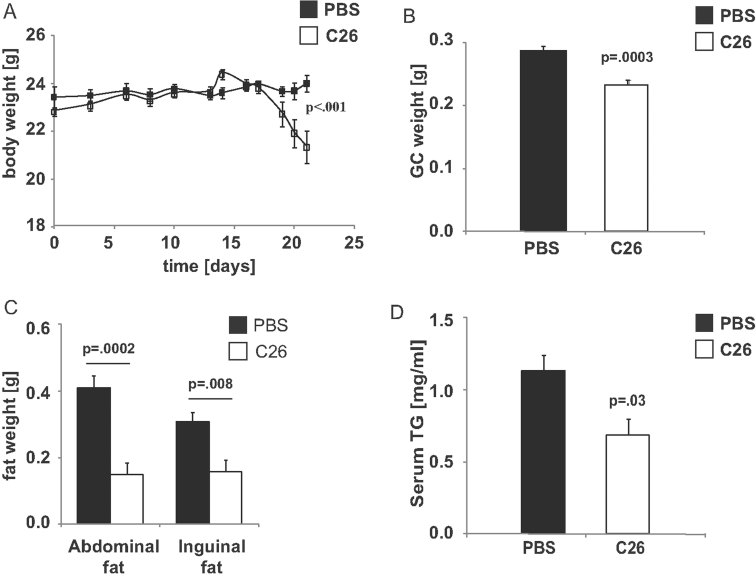
Serum triglycerides are lowered in cancer cachexia Body weight development of Balb/C mice treated with PBS or 1.5 × 10^6^ colon 26 (C26) cells over 3 weeks (means ± SEM, *n* ≥ 6, Two Way Repeated Measures ANOVA, Holm–Sidak *post hoc*).Gastrocnemius (GC) skeletal muscle weight in the same mice as in **A**.Abdominal and inguinal fat weight in the same mice as in **A**.Serum triglyceride levels in the same mice as in **A**. Statistical test **B**–**D**: Student's *t*-test.

We employed the well-established colon (C) 26 mouse model for tumour-induced cachexia to delineate the properties of hepatic energy homeostasis during cancer cachexia (Tanaka et al, 1990). To investigate the metabolic impact of tumour growth independently of changes in food intake (anorexia), the experiment was terminated when the tumour-bearing animals had lost roughly 10% of their initial body weight (Fig 1A). At this time point, subcutaneous implantation of C26 cells into wild-type mice (Supporting Information Fig S1A) promoted loss of skeletal muscle and adipose tissue mass, while food intake as well as hepatic cholesterol and triglyceride (TG) content remained unaltered (Fig 1B and C, Supporting Information Fig S1B–D). Circulating levels of non-esterified fatty acids (NEFA) also tended to be decreased in cachectic animals (unpublished observations).

Importantly, serum TG as the main systemic energy carriers were found to be significantly diminished in C26 mice as compared with controls (Fig 1D). In this respect, circulating serum TG levels are determined by the relative balance of hepatic very-low-density-lipoprotein (VLDL) production and release, peripheral TG hydrolysis by lipoprotein lipase (LPL) and subsequent clearance into adipose tissue and skeletal muscle, as well as receptor-mediated hepatic VLDL re-uptake (Postic & Girard, 2008).

**Figure 2:**
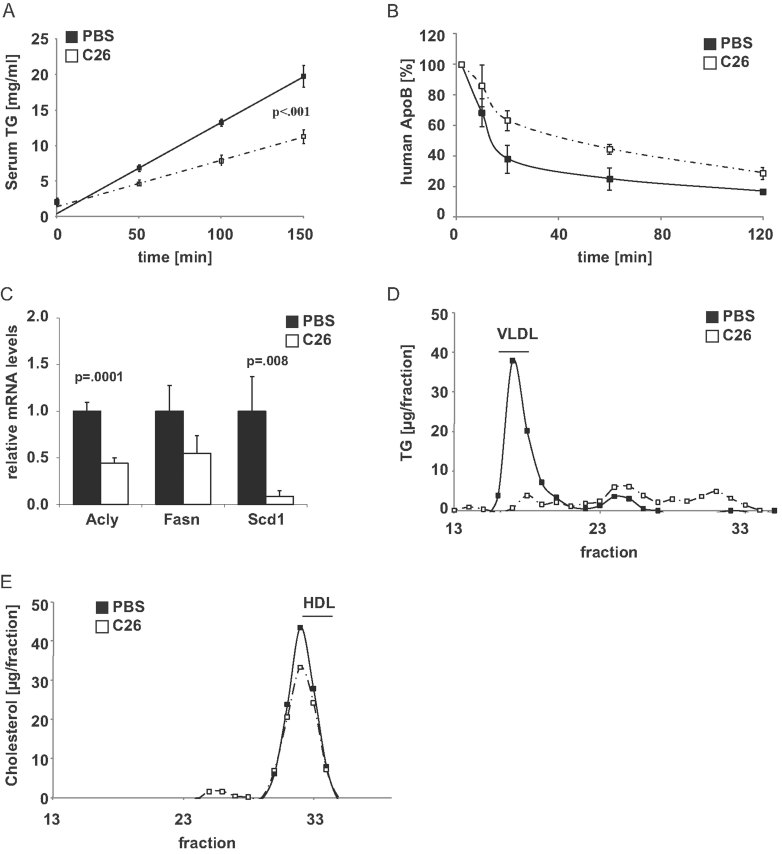
Cachectic mice display impaired hepatic VLDL secretion and hypobetalipoproteinemia Hepatic VLDL release in Balb/C mice injected either PBS or 1.5 × 10^6^ C26 cells (means ± SEM, *n* ≥ 6, Two Way Repeated Measures ANOVA, Holm-Sidak *post hoc*).Clearance of human ApoB from serum of Balb/C mice injected with either PBS or 1.5 × 10^6^ C26 cells. 20 µg of human VLDL were injected into each animal and serum samples were taken at the indicated time points. Human ApoB levels were determined by human-specific ELISA (means ± SEM, *n* = 2–4).Quantitative PCR analysis of hepatic ATP citrate lyase (Acly), fatty acid synthase (Fasn) and stearoyl-CoA desaturase-1 (Scd1) RNA levels in the same mice as in **A** (Student's *t*-test).Lipoprotein-associated serum TG levels as measured by fast protein liquid chromatography (FPLC) in the same mice as in **A**.Lipoprotein-associated serum cholesterol levels as measured by fast protein liquid chromatography (FPLC) in the same mice as in **A**.

Consistent with the hypothesis that cancer cachexia specifically disrupts the efficient mobilization and/or shift from intra-hepatic to serum TG pools, C26 mice showed significantly decreased hepatic VLDL secretion as compared with non-tumour-bearing littermates (Fig 2A), while hepatic ApoB uptake capacity from the serum was slightly impaired (Fig 2B). In congruence with the impaired hepatic VLDL secretion rate under cachectic conditions, tumour-bearing animals showed significant downregulation of genes in the lipogenic pathway in the liver as compared with healthy controls, thereby impairing substrate provision for the VLDL assembly pathway (Fig 2C; Elam et al, 2001; Moon et al, 2011).

Furthermore, fast protein liquid chromatography (FPLC)-mediated fractionation studies demonstrated the almost complete absence of VLDL-associated TG in the serum of tumour-bearing, cachectic animals (Fig 2D), while the cholesterol content of individual lipoprotein species remained unaltered as compared with healthy control animals (Fig 2E). Thereby, these experiments established experimental cancer cachexia as a state of peripheral energy depletion and loss of body mass, associated with hepatic VLDL hypo-secretion and non-familial hypobetalipoproteinemia.

### Cancer cachexia controls distinct molecular output pathways in the liver

The differential gene expression of specific transcriptional regulators between healthy and metabolic disease conditions has frequently been found to reflect a causal, functional role of these factors in the pathogenesis of severe metabolic disorders (Berriel Diaz et al, 2008; Herzig et al, 2001). The profound changes in hepatic lipid handling in tumour-bearing mice therefore prompted us to screen for the dysregulation of distinct transcriptional co-factor gene expression under cachectic conditions, exemplifying nuclear receptor, inflammatory and hormonal (glucocorticoid) signaling in liver.

**Figure 3:**
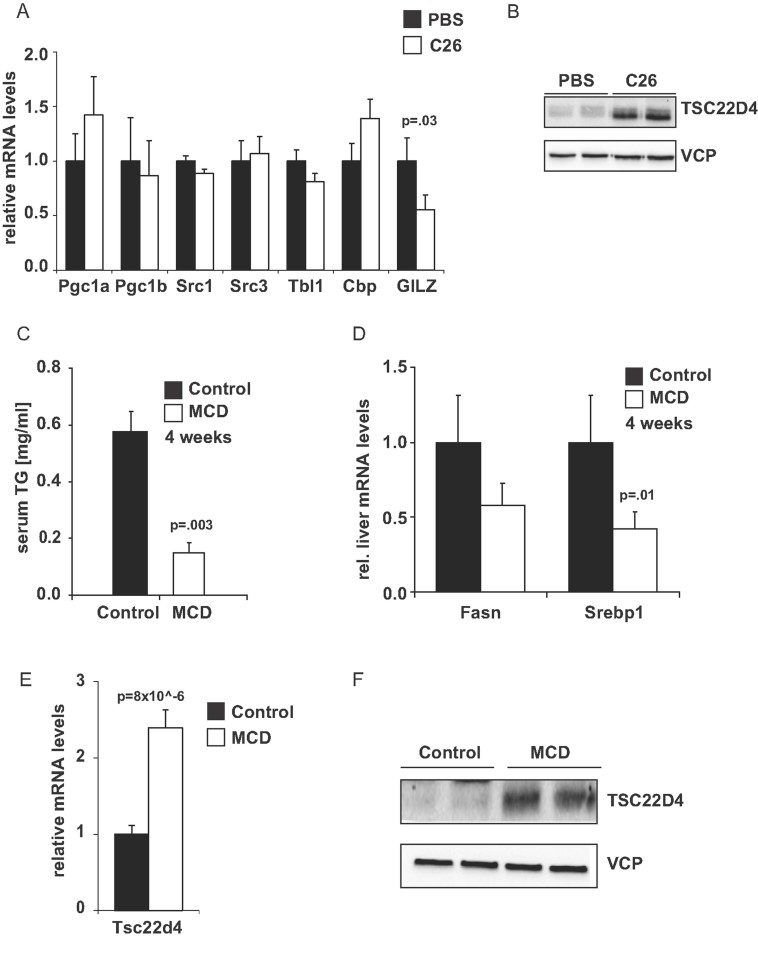
TSC22D4 is induced in experimental wasting conditions Quantitative PCR analysis of Peroxisome proliferator-activated receptor gamma coactivator 1-alpha (Pgc1a), Peroxisome proliferator-activated receptor gamma coactivator 1-beta (Pgc1b), Nuclear receptor coactivator 1 (Src1), nuclear receptor coactivator 3 (Src3), Transducin beta like 1 (Tbl1), Creb binding protein (Cbp) and Tsc22 domain family member 3 (Tsc22d3) in Balb/C mice injected with either PBS or 1.5 × 10^6^ C26 cells (means ± SEM, *n* ≥ 6). Statistical test: Student's *t*-test.Western blot analysis of TSC22D4 and valosine-containing protein (VCP) expression in representative mice from **A**.Serum TG levels in C57Bl/6 mice fed a methionine and choline deficient diet (MCD) or a corresponding control diet for 4 weeks (means ± SEM, *n* ≥ 5).Quantitative PCR analysis of fatty acid synthase (Fasn) and sterol regulatory element-binding protein-1c (Srebp1) RNA levels in the same mice as in **C**.Quantitative PCR analysis of Tsc22 domain family member 4 (Tsc22d4) RNA levels in the same mice as in **C**.Western blot analysis of TSC22D4 and VCP expression in representative mice from **C**. Statistical test **C**–**E**: Student's *t*-test.

Gene expression analysis revealed no obvious differences in mRNA levels for transcriptional co-factors PGC-1alpha/beta, SRC1, SRC3, TBL1 and CBP in cachectic livers, whilst GILZ was downregulated (Fig 3A). In contrast, both mRNA as well as protein levels of leucine zipper transcription factor TSC22D4 were found to be induced in livers of tumour-bearing, cachectic mice as compared with healthy control littermates (Fig 3B, Supporting Information Fig S2A), demonstrating that tumour-associated energy wasting is tightly and specifically associated with elevated TSC22D4 expression in the liver.

To extend these findings to an independent model, wild-type C57Bl/6 mice were placed on a methionine-choline-deficient (MCD) diet for 4 weeks, which is known to trigger peripheral hyper-metabolism and weight loss (Rizki et al, 2006; Supporting Information Fig S2B; Supporting Information Fig S2C) In addition, the liver TG content was elevated by ∼fivefold (Supporting Information Fig S2D), correlating with enhanced serum levels of liver damage markers (Supporting Information Fig S2E) upon MCD diet exposure. At the same time, serum TG levels, lipogenic gene expression, blood glucose levels and liver weight were significantly decreased (Fig 3C and D; Supporting Information Fig S2F, Supporting Information Fig S2G).

Consistent with the cachectic phenotype in tumour-carrying animals, levels of TSC22D4 were found to be significantly upregulated in livers of MCD-fed animals as compared to controls (Fig 3E). Furthermore, Western blot analysis demonstrated a marked induction also of TSC22D4 protein levels in MCD-treated animals (Fig 3F), suggesting that the induction of TSC22D4 expression represents a more global but distinct feature of wasting-associated liver metabolism.

In agreement with this assumption, TSC22D4 expression was significantly reduced in mice fed a high fat diet for 12 weeks, thereby displaying insulin resistance and diet-induced obesity (Supporting Information Fig S2H), overall suggesting that TSC22D4 is downregulated upon metabolic stress conditions, as associated with excess energy stores and availability.

### Liver TSC22D4 controls intra- and extra-hepatic TG levels

Transforming growth factor (TGF) beta 1-stimulated clone 22 (TSC22) D4 (also known as THG-1) was originally cloned based on sequence homologies with growth factor- and glucocorticoid-inducible TSC22 transcription factor family members D1 and D3, and subsequently was found to indeed posses transcriptional activity when fused to heterologous DNA binding domains (Kester et al, 1999). The biological and tissue-specific functions, and the target genes of TSC22D4 in the adult organism have not been defined to date.

**Figure 4:**
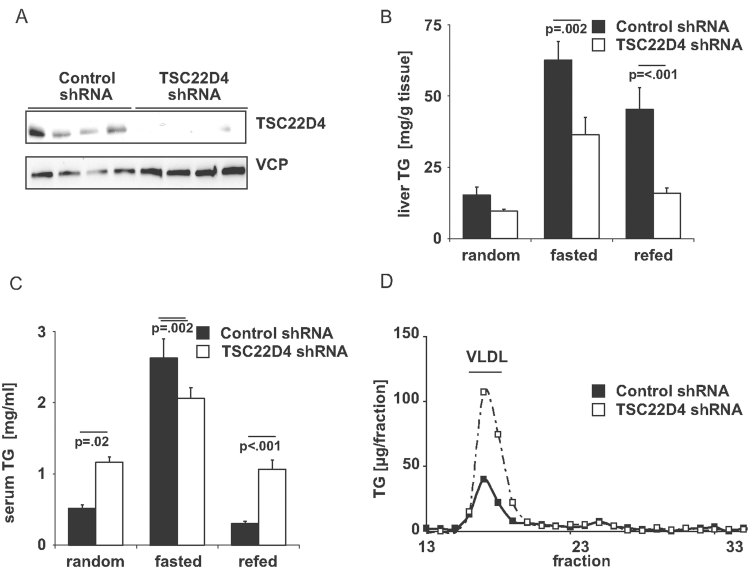
Hepatic TSC22D4 deficiency promotes hypertriglyceridemia Western blot of liver extracts from representative control or TSC22D4 shRNA adenovirus–injected C57Bl/6 mice 7 days after injection using TSC22D4 and VCP antibodies.Liver TG levels of random fed, 16 h fasted and 6 h refed control or TSC22D4 shRNA adenovirus–injected C57Bl/6 mice 7 days after injection (means ± SEM, *n* ≥ 6).Total serum TG levels of the same mice as in **B**.Lipoprotein-associated serum TG levels as measured by fast protein liquid chromatography (FPLC) in random fed control or TSC22D4 shRNA adenovirus-injected C57Bl/6 mice 7 days after injection (means ± SEM, *n* ≥ 6). Statistical test **B**, **C**: Two Way ANOVA; Holm–Sidak *post hoc*.

Thus, we first aimed at mimicking a TSC22D4-deficient metabolic status by liver-specific genetic manipulation. To this end, we disrupted the activity of TSC22D4 in livers of lean wild-type mice by delivering an adenovirus expressing a TSC22D4-specific or a non-specific control shRNA via tail vein injection. TSC22D4 shRNA treatment significantly reduced hepatic TSC22D4 protein levels as compared with control shRNA-injected littermates (Fig 4A). At day 7 after injection, acute knockdown of TSC22D4 caused no major differences in body-, liver-, total fat- and lean weight (Supporting Information Fig S3A–D), serum and hepatic cholesterol levels (Supporting Information Fig S3F and G), circulating and hepatic NEFA (Supporting Information Fig S3H and I), and serum insulin levels (Supporting Information Fig S3J), compared with controls. In contrast, loss of hepatic TSC22D4 resulted in a significant decrease in liver TG levels, which was evident upon both feeding and fasting conditions (Fig 4B), and a more than 2-fold elevation of total and (VLDL)-associated serum TG levels in the fed state (Fig 4C and D), indicating that TSC22D4 controls hepatic and systemic lipid handling in healthy wild-type animals.

To confirm the results from acute knockdown experiments in an independent setting, wild-type mice were injected with an adeno-associated virus (AAV) expressing a TSC22D4-specific miRNA or a non-specific control miRNA under the control of a hepatocyte-specific promoter (LP.1), allowing the expression of inhibitory miRNAs specifically in liver parenchymal cells but not in other liver cell types (Rose et al, 2011). After 2 weeks, TSC22D4 miRNA delivery led to a more than 60% decrease in hepatic TSC22D4 mRNA levels (Supporting Information Fig S3K) and also reduced TSC22D4 protein expression in liver as compared with control miRNA- or PBS-injected littermates (Supporting Information Fig S3L). Inactivation of TSC22D4 again triggered an increase in serum TG levels (Supporting Information Fig S3M), while leaving serum cholesterol levels unaffected (Supporting Information Fig S3N).

**Figure 5:**
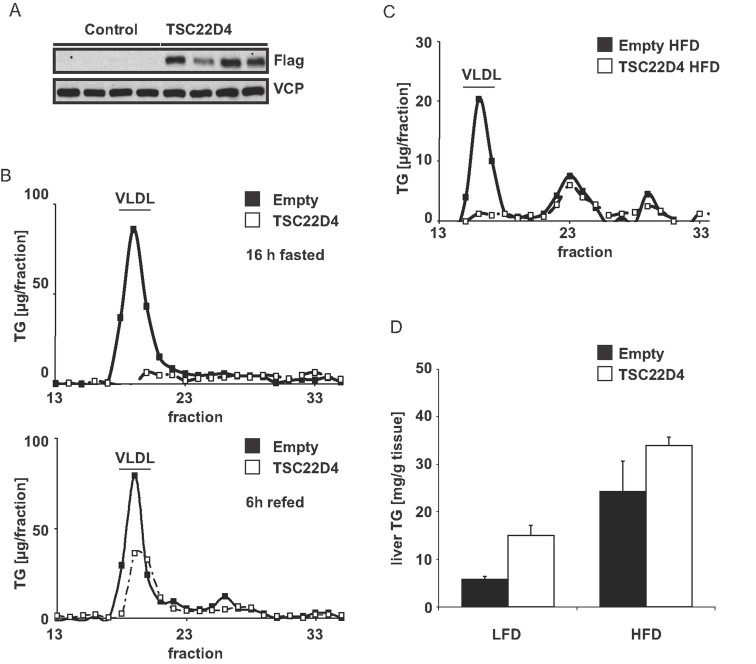
TSC22D4 overexpression reduces serum VLDL triglycerides Western blot of liver extracts from representative empty control or Flag-TSC22D4 cDNA adenovirus-injected C57Bl/6 mice 7 days after injection using FLAG and VCP antibodies (means ± SEM, *n* ≥ 4).Lipoprotein-associated serum TG levels as measured by fast protein liquid chromatography (FPLC) in mice injected with empty control or TSC22D4 cDNA adenovirus in the fed or 16 h fasted state (means ± SEM, *n* ≥ 4).Lipoprotein-associated serum TG levels as measured by fast protein liquid chromatography (FPLC) in high fat diet (HFD) fed mice injected with empty control or TSC22D4 cDNA adenovirus (means ± SEM, *n* ≥ 3).Liver TG levels of HFD or control diet (LFD) fed mice (means ± SEM, *n* ≥ 3, Two Way ANOVA; Holm–Sidak *post hoc*).

Given the substantial upregulation of TSC22D4 under conditions of energy wasting (Fig 3B and F), we next sought to test the effects of TSC22D4 overexpression (Fig 5A) on systemic lipid handling. To this end, we employed wild-type mice with adenovirus-mediated liver-specific overexpression of TSC22D4 in a fasting-feeding regimen. In line with the results from TSC22D4 loss-of-function experiments (Fig. 4), overexpression of TSC22D4 led to a substantial reduction of VLDL-associated serum TG levels, particularly under fasting conditions (Fig 5B), leaving other metabolic parameters mostly unaltered (Supporting Information Fig S4A–F). To next explore whether TSC22D4 over-activation could potentially even counteract hyper-triglyceridemia as associated with energy surplus, mice were placed on a high-fat diet (60% calories from fat vs. 10% calories from fat) for 12 weeks and then injected with an adenovirus carrying the TSC22D4 cDNA. Consistent with the results from acute TSC22D4 loss- and gain-of-function data, overexpression of TSC22D4 (Supporting Information Fig S4G) was sufficient to substantially reduce serum levels of VLDL-associated TG (Fig 5C) and tended to induce hepatic lipid accumulation (Fig 5D), even in livers of overweight mice, while leaving other metabolic parameters generally unaltered (Supporting Information Fig S4H–L). Together, these data underline the notion that hepatic TSC22D4 fulfils a critical and specific checkpoint function for the regulation of circulating VLDL-TG and hepatic TG load, and that high TSC22D4 levels induce serum TG depletion and hypobetalipoproteinemia under both normal and high caloric intake conditions.

### TSC22D4 regulates hepatic VLDL release and lipogenic gene expression

**Figure 6:**
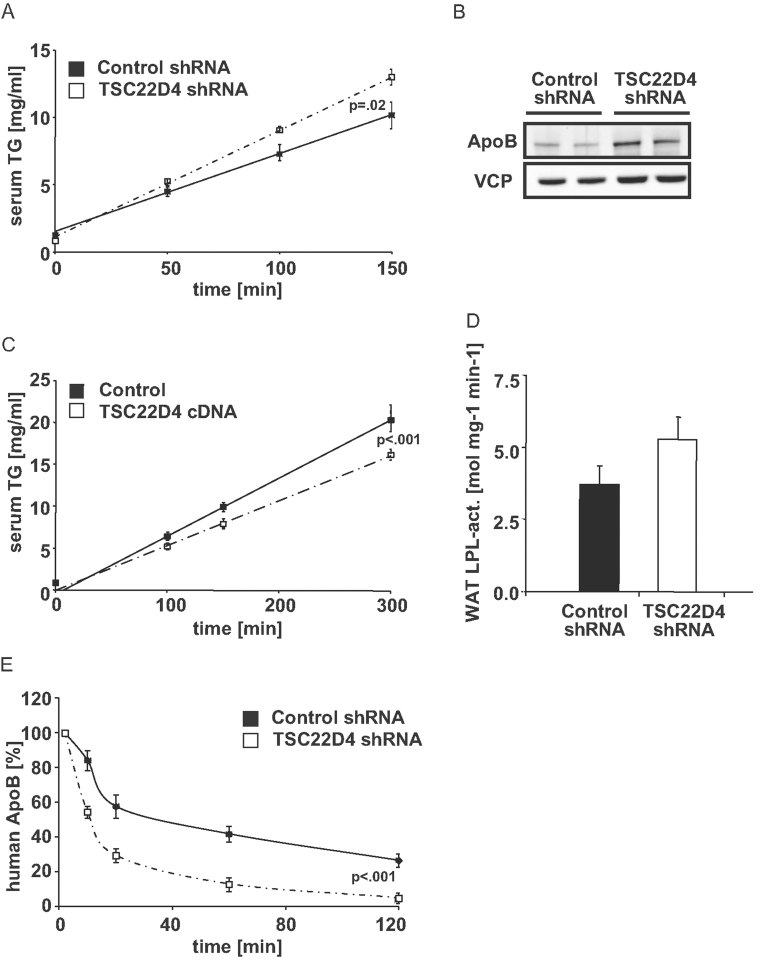
VLDL secretion is increased in TSC22D4 deficient mice Hepatic VLDL release in control or TSC22D4 shRNA-injected wild-type C57Bl/6 mice. Time after tyloxapol injection indicated (means ± SEM, *n* = 6). Statistical test: Two Way Repeated Measures ANOVA, Holm–Sidak *post hoc*.Apolipoprotein B protein expression is elevated in the livers of representative TSC22D4 shRNA adenovirus-injected C57Bl/6 mice. Western blots were performed 7 days after injection using ApoB and VCP antibodies.Hepatic VLDL release in empty control or TSC22D4 cDNA-injected wild-type C57Bl/6 mice fed a high fat diet (HFD) for 11 weeks. Time after tyloxapol injection indicated (means ± SEM, *n* = 4). Statistical test: Two Way Repeated Measures ANOVA, Holm–Sidak *post hoc*.White adipose tissue (WAT) lipoprotein lipase (LPL) activity in the same mice as in **A**. (student's *t*-test)Clearance of human ApoB from serum of control or TSC22D4 shRNA adenovirus–injected C57Bl/6 mice 7 days after virus injection. 20 µg of human VLDL were injected into each animal and serum samples were taken at the indicated time points. Human ApoB levels were determined by human-specific ELISA (means ± SEM, *n* = 6). Statistical test: Two Way Repeated Measures ANOVA, Holm–Sidak *post hoc*.

To determine the mechanistic basis for TSC22D4 function in hepatic lipid handling, we measured hepatic VLDL production by experimental inhibition of peripheral VLDL clearance. Consistent with higher serum TG levels, mice deficient in hepatic TSC22D4 showed an increase in hepatic VLDL release as compared with controls (Fig 6A), correlating with enhanced levels of intra-hepatic ApoB as demonstrated by Western blot experiments (Fig 6B). In contrast, liver-specific overexpression of TSC22D4 significantly impaired hepatic VLDL release under high fat diet conditions (Fig 6C), overall suggesting that hepatic TSC22D4 controls systemic TG levels primarily through the control of liver lipid release. Indeed, adipose tissue LPL activity was not changed and VLDL transporter/receptor gene expression remained unaffected upon hepatic TSC22D4 deficiency (Fig 6D, Supporting Information Fig S5A). Hepatic VLDL uptake was actually slightly improved, perhaps as a compensatory response (Fig 6E).

**Figure 7:**
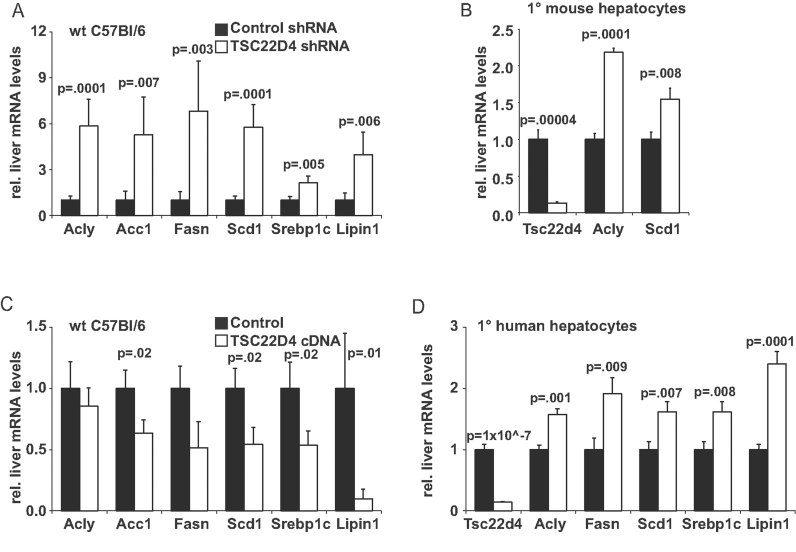
TSC22D4 regulates lipogenic genes in murine and human hepatocytes Quantitative PCR analysis of ATP citrate lyase (Acly), acetyl-coenzyme A carboxylase 1 (Acc1), fatty acid synthase (Fasn), stearoyl-CoA desaturase-1 (Scd1), sterol regulatory element-binding protein-1c (Srebp1) and Lipin1 RNA levels in livers of control or TSC22D4 shRNA adenovirus-injected wild-type C57Bl/6 mice (means ± SEM, *n* > 7).Quantitative PCR analysis of TSC22D4, Acly and Scd1 mRNA levels in primary (1°) mouse hepatocytes treated with control or TSC22D4 shRNA adenovirus (means ± SEM, *n* = 3).Quantitative PCR analysis of Acly, Acc1, Fasn, Scd1, Srebp1 and Lipin1 RNA levels in livers of control or TSC22D4 cDNA adenovirus-injected wild-type C57Bl/6 mice (means ± SEM, *n* > 4).Quantitative PCR analysis of Tsc22d4, Acly, Fasn, Scd1, Srebp1 and Lipin1 RNA levels in primary (1°) human hepatocytes treated with control or TSC22D4 shRNA adenovirus (means ± SEM, *n* = 3). Statistical test **A**–**D**: Student's *t*-test.

As tissue-specific target gene networks of TSC22D4 have not been investigated to date, we next sought to explore the molecular basis for TSC22D4 function in hepatic lipid homeostasis *in vivo*. In congruence with a stimulatory function of TSC22D4 deficiency for hepatic VLDL release, high throughput gene expression profiling revealed that TSC22D4 knockdown in wild-type mice induced the expression of key genes in the lipogenic pathway, including fatty acid synthase (FAS), ATP citrate lyase (ACLY) and sterol regulatory element binding protein (SREBP)-1c, as well as LIPIN1, a gene involved in hepatic VLDL production (Fig 7A). Bile acid synthesis, lipid transporter, as well as LPL inhibitor gene expression were however left unaltered as compared to controls (Supporting Information Fig S5B and C). Indeed, inactivation of TSC22D4 in primary mouse hepatocytes by shRNA-mediated gene knockdown resulted in a significant induction of genes in the fatty acid biosynthesis and biosynthesis of unsaturated fatty acids KEGG pathways (Fig 7B; Supporting Information Table 1), confirming the regulatory impact of TSC22D4 on lipid-generating gene networks in a cell autonomous manner.

Conversely, overexpression of TSC22D4 in livers of wild-type animals substantially impaired lipogenic gene expression in the fed state (Fig 7C), which was consistent with the inhibitory impact of TSC22D4 on hepatic VLDL release (Fig 6A) and further supported the notion that TSC22D4 represents a potent inhibitor of lipogenic pathways in the liver. In addition, TSC22D4 overexpression significantly reduced the activity of a FAS reporter gene in Hepa1c1 hepatocytes in a dose-dependent manner (Supporting Information Fig S5D), further indicating that TSC22D4 represents a critical transcriptional checkpoint for the lipogenic genetic pathway and the subsequent regulation of serum TG homeostasis via control of hepatic VLDL release. Importantly, TSC22D4-mediated regulation of lipogenic gene expression was also confirmed in human primary hepatocytes (Fig 7D), thereby demonstrating that TSC22D4-dependent lipid handling represents a conserved feature between both murine and human hepatocytes.

### Hepatic TSC22D4 associates with distinct features of cachectic liver metabolism

**Figure 8:**
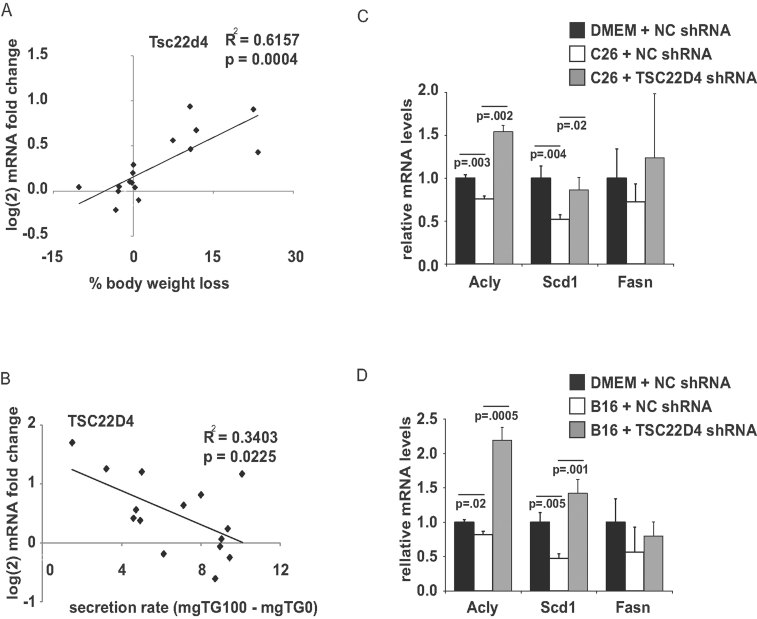
TSC22D4 expression correlates with the degree of body wasting Correlation of TSC22D4 RNA expression and the degree of weight loss due to tumour implantation in Balb/C mice treated with PBS or 1.5 × 10^6^ colon 26 (C26) cells over 3 weeks (means ± SEM, *n* ≥ 6, Pearson correlation coefficient, *F*-test to determine significance).Correlation of TSC22D4 RNA expression and VLDL-TG release in the same mice as in A. (Pearson correlation coefficient, *F*-test to determine significance)Quantitative PCR analysis of Acly, Scd1 and Fasn RNA levels in primary mouse hepatocytes treated with conditioned media from C26 cells for 24 h and control or TSC22D4 shRNA adenovirus for 48 h (means ± SEM, *n* = 3, Student's *t*-test for DMEM NC shRNA vs. C26 NC shRNA; C26 NC shRNA vs. C26 TSC22D4 shRNA).Quantitative PCR analysis of Acly, Scd1 and Fasn RNA levels in primary mouse hepatocytes treated with conditioned media from B16 cells for 24 h and control or TSC22D4 shRNA adenovirus for 48 h (means ± SEM, *n* = 3, Student's *t*-test for DMEM NC shRNA vs. B16 NC shRNA; B16 NC shRNA vs. B16 TSC22D4 shRNA).

The induction of TSC22D4 expression in cancer cachexia (Fig 3B) and its regulatory impact on hepatic lipid handling finally prompted us to correlate the hepatic expression levels of various transcriptional regulators, including TSC22D4, with either the degree of cachexia or hepatic VLDL release in tumour-bearing and healthy control animals. While these studies revealed a correlation between the expression levels of PGC-1alpha and TBL1 and wasting, PGC-1beta, SRC-1, SRC-3 and CBP mRNA levels did not correlate with tumour-induced body weight loss (Supporting Information Fig S6A and B). In contrast, TSC22D4 mRNA levels positively correlated with the degree of body weight loss and negatively correlated with hepatic VLDL secretion in tumour bearing versus control animals (Fig 8A and B). In line with the assumption that TSC22D4 mediates parts of the metabolic liver phenotype during cancer cachexia, inhibition of lipogenic gene expression in primary hepatocytes by cachexia-inducing conditioned C26 or B16 melanoma tumour cell supernatants was rescued by TSC22D4 knockdown (Fig 8C and D). These data overall indicated that TSC22D4 expression levels may serve as a specific molecular indicator of the hepatic lipid handling status particularly under conditions of energy deficiency, including tumour-induced cachexia.

## DISCUSSION

Our study identifies TSC22D4 as a cachexia-inducible regulator of hepatic VLDL secretion and a molecular determinant of circulating TG levels, associated with a regulatory function in the control of lipogenic gene expression in the liver.

Based on previous expression screening, TSC22D4 thus far had been implicated in pituitary development (Fiorenza et al, 2001) and in the adaptation of renal cells to hypertonicity. In this respect, TSC22D4 was found to be upregulated upon hyperosmolality in kidney cells, and overexpression of TSC22D4 in medullary collecting duct cells promoted cell survival upon osmotic stress in cell culture systems (Fiol et al, 2007). However, little further information about the biological function of TSC22D4 has so far been presented.

Our data now provide first insights into tissue-specific functions of the TSC22D4 protein with further implications for the pathogenesis of cancer-related cachexia. The results of our study are consistent with a model, in which tumour stress triggers the upregulation of hepatic TSC22D4 gene expression. TSC22D4 activation, in turn, is sufficient to impair hepatic lipogenesis and to prevent the mobilization of hepatic TG stores into the serum compartment by decreasing hepatic VLDL release, thereby establishing systemic hypo-triglyceridemia and hypobetalipoproteinemia as a hallmark of cancer cachexia and wasting-related metabolic stress conditions.

Indeed, impairment of hepatic VLDL release has been associated with peripheral energy depletion and catabolic wasting conditions, reflecting the importance of VLDL-associated TG and its lipoprotein component ApoB100 as both an energy source and signaling molecules for peripheral organs, respectively (Seelaender et al, 1996). Also, inhibition of hepatic ApoB expression correlates with the loss of adipose tissue mass (Mullick et al, 2011), and exposure of adipocytes to ApoB100 inhibits catecholamine-induced lipolysis in these cells (Skogsberg et al, 2008). This supports the idea that hepatic VLDL secretion does not only determine intra-hepatic TG stores and eventually hepatic lipid accumulation upon its impairment, but also serves as a circulating signal and energy substrate for peripheral tissues. Along these lines, hepatic TSC22D4 levels were indeed found to significantly correlate with the degree of body weight loss upon tumour development in this study. Consistent with previous reports indicating an important, as yet less appreciated, contribution of dysfunctional liver metabolism to a cachectic phenotype (Martignoni et al, 2009), these findings underline the hypothesis that specific intra-hepatic transcriptional programs significantly impact overall systemic energy availability and thereby further propagate an energy-deficient wasting condition in response to tumour burden.

The TSC22 domain family of transcriptional regulators is conserved from *Caenorhabditis elegans* to humans and is encoded by four separate genetic loci in mammals, referred to as TSC22D1 to TSC22D4 (Gluderer et al, 2010). TSC22D4 was originally identified as a member of the TSC22 protein family based on its homology to and interaction capabilities with TSC22D1 in an unbiased interactome screen. Consistent with the strong inhibitory effect of TSC22D4 on lipogenic gene expression in livers, transcriptional reporter assays documented potent transcriptional repressor activity when fused to heterologous DNA binding domains (Kester et al, 1999). Of note, the strong activating effect of TSC22D4 knockdown on LIPIN gene expression is consistent with the reported functional role of LIPIN as a cytosolic phosphatidic acid phosphatase promoting hepatic lipogenesis, hepatic VLDL secretion, and hepatic insulin resistance. Indeed, induction of the beta isoform of LIPIN through inhibition of the SFRS10 splicing factor gene in liver triggers increased hepatic VLDL secretion (Pihlajamaki et al, 2011), and LIPIN overexpression disturbs the insulin-signaling cascade (Ryu et al, 2009), which is consistent with the observed TSC22D4-mediated VLDL secretion phenotype. Whether the observed positive regulatory effect of TSC22D4 on other target gene pathways in genome-wide expression analyses also reflects a transcriptional activator function of TSC22D4 in specific promoter contexts, or whether it is indirectly mediated through the control of transcriptional repressor activity, awaits further clarification in the future.

The hepatic induction of TSC22D4 and its correlation with body weight loss in the tumour-bearing state is intriguing, as TSC22D4 belongs to a family of suspected tumour suppressor genes (Kester et al, 1999). Indeed, TSC22 family members have been shown in *Drosophila* to be part of a growth-promoting signaling complex involving the Mlf1 adapter molecule Madm (Gluderer et al, 2010), and a TSC22D1/D4 complex has been implicated in BRAF-induced senescence and neoplasia (Homig-Holzel et al, 2011). Furthermore, TSC22D1 was recently found to antagonize Ras/Raf signaling, thereby exerting anti-tumourigenic effects in a Ras/Raf-dependent liver cancer model (Nakamura et al, 2011), and TSC22D1 abrogated TGF-beta-induced growth rates in intestinal epithelial cells (Gupta et al, 2003). While direct pro- or anti-tumourigenic actions of TSC22D4 have not been investigated to date, the *Drosophila* homologue of TSC22D4 has been implicated in growth regulatory circuits (Gluderer et al, 2010), and ectopic expression of TSC22D4 abrogated proliferative arrest induced by BRAFE600 in melanocytes (Homig-Holzel et al, 2011). Given the metabolic control function of TSC22D4 as demonstrated by the present study, it is tempting to speculate that TSC22 proteins may represent a family of transcriptional regulators that is broadly involved in the control of proliferative and metabolic pathways during tumour development. It is tempting to speculate that the regulation of TSC22D4 expression is sensitive to certain metabolites, including amino acids, as one-carbon unit deficiency may be a common feature between the MCD and C26 tumour models. Indeed, exogenous supply of the one-carbon unit donor methionine has been able to reverse distinct features of cancer-induced weight loss in human patients (Sengelov et al, 1994). The regulation of TSC22D4 in response to tumour stress and its implications in liver cell metabolism might thereby exemplify a common transcriptional node in the documented link between obesity-related dyslipidemia and an increased risk for certain cancer entities (Grote et al, 2010). As disturbances in systemic lipid metabolism, including the loss of phospholipid FA, have been shown to correlate with the survival of advanced cancer patients (Murphy et al, 2010), our findings clearly validate future studies in this direction.

## MATERIALS AND METHODS

### Recombinant viruses

Adenoviruses expressing a TSC22D4 or a non-specific shRNA under the control of the U6 promoter, or the TSC22D4 cDNA under the control of the CMV promoter were cloned using the BLOCK-iT Adenoviral RNAi expression system (Invitrogen, Karlsruhe, Germany). Viruses were purified by the cesium chloride method and dialysed against phosphate-buffered-saline buffer containing 10% glycerol prior to animal injection, as described previously (Herzig et al, 2001, 2003). AAVs encoding control or TSC22D4-specific miRNAs under the control of a hepatocyte-specific promoter were established as described previously (Rose et al, 2011).

### Animal experiments

Male 8–12 week old C57Bl/6 and Balb/C mice were obtained from Charles River Laboratories (Brussels, Belgium) and maintained on a 12 h light–dark cycle with regular unrestricted diet. For hepatic VLDL release and fasting experiments, animals were fasted for 16 h. Otherwise, animals were fed *ad libitum* and had free access to water. For adenovirus injections, 1–2 × 10^9^ plaque-forming units (pfu) per recombinant virus were administered via tail vein injection. For AAV experiments, 5 × 10^11^ viruses were injected via the tail vein. For tumour induction in cachexia models, 1.5 × 10^6^ C26 cells in phosphate buffered saline were injected subcutaneously into 10-week-old Balb/C mice (Charles River Laboratories, Brussels, Belgium). In high-fat diet experiments, C57Bl/6 mice were either fed a standard chow diet (10% energy from fat, Research diets D12450B, New Brunswick, USA) or a high-fat diet (60% energy from fat, Research diets D12492) for a period of 12 weeks. For MCD diet experiments, C57Bl/6 mice were either fed a standard chow diet or a methionine, choline deficient (MCD) diet (Research diets) for a period of 4 weeks. In each experiment, 4–10 animals received identical treatments and were analysed under fasted or fed conditions as indicated. Organs including liver, kidney, epididymal fat pads, and gastrocnemius muscles were collected after specific time periods, weighed, snap-frozen and used for further analysis. Total body fat content was determined by an Echo MRI body composition analyser (Echo Medical Systems, Houston, USA). Animal handling and experimentation was done in accordance with NIH guidelines and approved by local authorities.

### Experiments involving human subjects

Experiments involving human subjects were carried out according to the WMA Declaration of Helsinki 2008 and the NIH Belmont Report. Informed consent was obtained from all subjects.

### Blood metabolites

Serum levels of glucose, triglycerides (TG), cholesterol, total ketone bodies, and NEFA were determined using an automatic glucose monitor (One Touch, Lifescan, Neckargemünd, Germany) or commercial kits (Sigma, Munich, Germany; RANDOX, Crumlin, Northern Ireland; WAKO, Neuss, Germany, respectively). Insulin levels were determined using a mouse insulin enzyme-linked immunosorbent assay (Mercodia, Uppsala, Sweden).

### Fast protein liquid chromatography

Serum from 4 to 10 mice per experimental group was pooled and subjected to fast protein liquid chromatography as previously described (Lichtenstein et al, 2007). Cholesterol and TG levels were measured in the eluted fractions using commercial kits as above.

### Hepatic VLDL release

VLDL production was determined after tyloxapol (Sigma, Munich, Germany) injection as described (Mandard et al, 2006).

### Human VLDL clearance

Human VLDL was isolated from fasting serum samples by ultracentrifugation as described (Redgrave et al, 1975). Briefly, 3.5 ml serum was placed in a SW40Ti polyallomer tube and mixed with 1.39 g KBr, overlayered with 332.8 ml of a NaCl/KBr solution (*D* = 1.063, 1.019 and 1.006 g/ml) and run for 18 h at 40,000 rpm. Human VLDL (20 mg) was injected into each animal, and serum samples were taken at 2, 10, 30, 60 and 120 min. Serum human ApoB-100 levels were measured using a human-specific ApoB ELISA. For the ELISA, we used a primary coating antibody generated against human apoB-100 (mAb47, kindly supplied by J. Witztum, University of San Diego, USA), in a concentration of 2 mg/well IgG protein in TBS/EDTA/BHT and a secondary biotinylated polyclonal antibody raised in goat against human ApoB in a concentration of 4 ug/well in 1.5% BSA/TBS/0.1% Tween. To prevent non-specific binding, plates were blocked with 1.5% BSA/TBS/0.1% Tween. Samples were diluted 1:25. Absorbance was read 30 min after addition of TMB and termination of the reaction with 2 M H_2_SO_4_ at 450 nm (Groot et al, 1991).

### LPL activity

LPL activity measurements were performed as described (Klingenspor et al, 1989) using frozen adipose tissue samples.

### Tissue lipid extraction

Hepatic lipids were extracted as previously described (Herzig et al, 2003), and TG and total cholesterol content were determined using commercial kits as above. Values were calculated as mg (TG and cholesterol) per gram wet tissue.

### Histochemistry

Liver tissue was embedded in Tissue Tek optimal cutting temperature compound (Sakura, Torrance, USA). Five-micrometer cryosections were stained with haematoxylin and eosin or Oil Red O as described (Peet et al, 1998).

### Quantitative Taqman RT-PCR

Total RNA was extracted from homogenized mouse liver or cell lysates using Qiazol reagent (Qiagen, Hilden, Germany). cDNA was prepared by reverse transcription using the M-MuLV enzyme and Oligo dT primer (Fermentas, St. Leon-Rot, Germany). cDNAs were amplified using assay-on-demand kits and an ABIPRISM 7700 Sequence detector (Applied Biosystems, Darmstadt, Germany). RNA expression data was normalized to levels of TATA-box binding protein (TBP) RNA.

### Protein analysis

Protein was extracted from frozen organ samples or cultured hepatocytes in cell lysis buffer (Rose et al, 2007) and 20 µg of protein were loaded onto 4–12% SDS-polyacrylamide gels and blotted onto nitrocellulose membranes. Western blot assays were performed as described (Herzig et al, 2001) using antibodies specific for TSC22D4 (Abcam, Cambridge, UK or Sigma, Munich, Germany), FLAG peptide (Santa Cruz Antibodies, Santa Cruz, USA), LIPIN1 (Abcam, Cambridge, UK), ACLY (Abcam), APOB (Santa Cruz Antibodies) or VCP (Abcam).

### Plasmids and RNA interference

For shRNA experiments, oligonucleotides targeting mouse TSC22D4 (GCCTGGTTGGCATTGACAACACGAATG), were annealed and cloned into the pENTR/U6 shRNA vector (Invitrogen). Non-specific oligonucleotides (5′-GATCTGATCGACACTGTAATG-3′) with no significant homology to any mammalian gene sequence were used as non-silencing controls in all experiments. For miRNA experiments, oligonucleotides targeting mouse TSC22D4 (5′-GACAGCGATGACGATAGTGGT-3′) and non-specific oligonucleotides (5′-AAATGTACTGCGCGTGGAGAC-3′) were cloned into the pdsAAV-LP1 vector. The FasN-Luc reporter plasmid was kindly provided by Timothy Osborne (UCI). Expression vectors for TSC22D4 were generated by standard PCR-based methods and cloned into the pcDNA3.1 expression vector (Promega, Mannheim, Germany).

**The paper explained**

PROBLEM:

Cancer-induced cachexia is characterized by massive loss of adipose tissue and skeletal muscle mass, and is believed to lead up to 30% of cancer-related deaths in humans. Currently, there are no effective preventive or therapeutic measures against cancer cachexia, most likely reflecting the heterogeneity and combinatorial action of host- and/or tumour-derived mediators that ultimately determine the systemic cachectic response. The definition of specific molecular mechanisms in affected target tissues may pave the way to novel therapeutic approaches in these patients, but still remains vastly incomplete.

RESULTS:

Our study explores the metabolic features of liver metabolism during experimental cancer cachexia. We demonstrate that the provision of systemic energy substrates through the liver is greatly impaired in the tumour-bearing condition, mediated via the inhibition of hepatic VLDL lipid secretion. At the molecular level, the gene-regulatory protein TSC22D4 is identified as a novel molecular output pathway of cachectic liver metabolism, which controls hepatic VLDL release and the activity of lipid-generating pathways. Indeed, hepatic levels of TSC22D4 correlate with the degree of tumour-induced body weight loss and energy deficiency in cachectic animals.

IMPACT:

Our findings underline the hypothesis that specific molecular programs in the liver significantly impact on overall systemic energy availability and thereby further promote an energy-deficient state in response to tumour development.

Given the control function of TSC22D4 for hepatic lipid homeostasis, TSC22D4 may represent a family of molecular regulators that explains parts of the peripheral energy deprivation in patients with cancer cachexia. Indeed, essential components of hepatic TSC22D4 function are conserved between mice and men, further underlining the importance of our findings for the human situation and suggesting the future exploitation of TSC22D4 as a therapeutic candidate in anti-cachexia therapies. Overall, our findings may help to shift the focus of current clinical cachexia research from classical (skeletal muscle, adipose tissue) to non-classical (liver) target organs as critical effectors of systemic energy wasting.

### Cell culture and transient transfection assays

Hepa1c1 cells were transfected using (PEI) reagent as described (Reed et al, 2006). Cell extracts were prepared 48 h after transfection, and luciferase assays were performed as described (Herzig et al, 2001), normalizing to the activity from a co-transfected β-galactosidase expression plasmid. Primary mouse hepatocytes were isolated and cultured as described (Klingmuller et al, 2006). Briefly, male 8–12 week old C57Bl/6 mice were anaesthetized by i.p. injection of 100 mg/kg body weight ketamine hydrochloride and 5 mg/kg body weight xylazine hydrochloride. After opening the abdominal cavity, the liver was perfused at 37°C with HANKS I (8 g NaCl, 0.4 g KCl, 3.57 g Hepes, 0.06 g Na_2_HPO_4_ × 2 H_2_O, 0.06 g KH_2_PO_4_ in 1 L distilled H_2_O, 2.5 mM EGTA, 0.1% glucose, adjusted to pH 7.4) via the portal vein for 5 min and subsequently with HANKS II (8 g NaCl, 0.4 g KCl, 3.57 g Hepes, 0.06 g Na_2_HPO_4_ × 2 H_2_O, 0.06 g KH_2_PO_4_ in 1 L distilled H_2_O, 0.1% glucose, 3 mg/ml collagenase CLSII, 5 mM CaCl_2_, adjusted to pH 7.4) for 5–7 min until disintegration of the liver structure was observed. The liver capsule was removed and the cell suspension was filtered through a 100 µm mesh. The cells were washed and, subsequently, viability of cells was determined by trypan blue staining. 1 000 000 living cells/well were seeded on collagen I-coated six-well plates. After 24 h, cells were infected with recombinant adenoviruses at a multiplicity of infection of 100. For stimulation experiments, primary hepatocytes were treated with control medium or conditioned supernatant that had previously been incubated with C26 colon cancer or B16 melanoma cells for 48 h. Conditioned media were applied to primary hepatocytes for 24 h prior to harvest. Primary human hepatocytes were obtained from Promocell, Heidelberg and cultivated according to the company's protocol. 24 h after seeding, cells were infected with recombinant adenoviruses at a multiplicity of infection of 100. Cells were harvested 48 h after infection.

### Metabolic flux analysis

Mitochondrial activity was determined using an XF96 Extracellular Flux Analyser (Seahorse, Copenhagen) and the corresponding XF Cell Mito Stress Test kit. Prior to the experiment, 20 000 Hepa1c1 cells were seeded per well in a 96-well format. The cells were subsequently incubated with control medium or conditioned medium (C26 or B16) for 24 h. The assay was performed according to the manufacturer‘s protocol using 2 µM Oligomycin, 0.5 µM FCCP, 1 µM Rotenone and 1 µM Antimycin A. Results were normalized to intracellular protein content, as determined by Sulforhodamine B staining.

### Gene expression profiling

Gene expression profiling was performed on liver extracts from control or TSC22D4 knockdown mice and primary hepatocytes treated with control or TSC22D4 shRNA adenovirus. RNA isolation, cDNA and cRNA synthesis, and hybridization to arrays of type Mouse Genome 430 2.0 from Affymetrix was performed according to the manufacturer's recommendations. Three arrays per genotype were hybridized. Microarray data were analysed based on ANOVA using a commercial software package (Micro Array Solution, version 1.0; SAS Institute, Cary, NC). Standard settings were used, except for the following specifications: log-linear mixed models were fitted for values of perfect matches, with genotype considered to be constant and the array identification, random. Custom CDF with Unigene-based gene/transcript definitions was used to annotate the arrays. Affected biological pathways reflected by the differential gene expression were determined by ORA based on Fisher's exact test.

### Statistical analysis

For each experiment, means ± SEM were determined. Statistical analyses were performed using student's *t*-test in one-factorial designs. Correlation was determined using Pearson's correlation coefficient; *F*-test was applied to determine significance. For multifactorial study designs, Two-way ANOVA and two-way ANOVA RM were used when appropriate. Holm–Sidak *post hoc* was applied when significant differences were found with an overall significance level = 0.05.

## Supplementary material

As a service to our authors and readers, this journal provides supporting information supplied by the authors. Such materials are peer reviewed and may be re-organized for online delivery, but are not copy-edited or typeset. Technical support issues arising from supporting information (other than missing files) should be addressed to the authors.
