# Corrigendum

**DOI:** 10.1002/emmm.201390008

**Published:** 2013-06-04

**Corrigendum to** Jones A, Friedrich K, Rohm M, Schäfer M, Algire C, Kulozik P, Seibert O, Müller-Decker K, Sijmonsma T, Strzoda D, Sticht C, Gretz N, Dallinga-Thie GM, Leuchs B, Kögl M, Stremmel W, Berriel Diaz M, and Herzig S (2013) TSC22D4 is a molecular output of hepatic wasting metabolism. EMBO Mol Med 5 (294–308) DOI: 10.1002/emmm.201201869

The authors of the above research article have informed the journal that an error occurred with respect to acknowledgement of funding. The originally acknowledged funding of the Deutsche Forschungsgemeinschaft granted to Stephan Herzig is not related to the research presented here. The correct reference number of the grant supporting work related to the article is HE3260/7-1.

The authors apologize for any confusion.

