# Supplementary material for: TSC22D4 is a molecular output of hepatic wasting metabolism
Source: EMBO Mol Med. 2013 Jan 11;5(2):294–308. doi: 10.1002/emmm.201201869 (PMC3569644; doi:10.1002/emmm.201201869)
Supplement: Supplementary file 1 [file emmm0005-0294-SD1.pdf]

EMBO Molecular Medicine

TSC22D4 is a molecular output of hepatic wasting metabolism

Allan Jones, Kilian Friedrich, Maria Rohm, Michaela Schäfer, Carolyn Algire, Philipp Kulozik, Oksana Seibert, Karin Müller-Decker, Tjeerd Sijmonsma, Daniela Strzoda, Carsten Sticht, Norbert Gretz, Geesje Dallinga-Thie, Barbara Leuchs, Manfred Kögl, Wolfgang Stremmel, Mauricio Berriel Diaz and Stephan Herzig

Corresponding author: Stephan Herzig, German Cancer Research Center

Review timeline:

Submission date:	13 August 2012
Editorial Decision:	01 October 2012
Revision received:	12 October 2012
Editorial Decision:	22 October 2012
Revision received:	05 November 2012
Accepted:	16 November 2012

Transaction Report:

(Note: With the exception of the correction of typographical or spelling errors that could be a source of ambiguity, letters and reports are not edited. The original formatting of letters and referee reports may not be reflected in this compilation.)

1st Editorial Decision 01 October 2012

Thank you for the submission of your manuscript "Transforming growth factor-beta1 Stimulated Clone-22 D4 is a molecular output of hepatic wasting metabolism" to EMBO Molecular Medicine and please accept my apologies for the delayed reply. We have now heard back from the referees who agreed to evaluate your manuscript. You will see that they find the topic of your manuscript potentially interesting. However, they also raise significant concerns on the study, which should be addressed in a major revision of the manuscript.

In particular, reviewer #2 highlights that pair feeding studies have to be done and that tumor burden should be mentioned.

Given the balance of these evaluations, we would welcome a revision of your manuscript if you can convincingly address the issues that have been raised within the time constraints outlined below.

Revised manuscripts should be submitted within two months of a request for revision. They will otherwise be treated as new submissions, unless arranged otherwise with the editor.

I look forward to seeing a revised form of your manuscript as soon as possible.

Yours sincerely,

Editor
EMBO Molecular Medicine

***** Reviewer's comments *****

Referee #1 (Remarks):
It's an excellent work carried out by a highly qualified group of scientists. Although it is an exclusively experimental study it could be potentially translated, albeit not in the short term, into new therapeutic approaches for the treatment of cancer cachexia in the clinical practice.

Referee #2 (Comments on Novelty/Model System):

the lack of pair feeding studies make it unclear as to whether the alterations described are "tumor - induced" at all.

Referee #2 (Remarks):

Jones et al.
Introduction a broad ramble around various topics; I suggest that this section be completely rewritten with a specific emphasis on the topic of hepatic [lipid] metabolism in cancer cachexia, and culminationg in a statement of hypothesis of the work.

Authors clearly demonstrate a deficit in VLDL formation in murine cancer [colon26] cachexia as well as establishing TSC22D4 as a key regulatory point in hepatic lipid metabolism
Completely new findings on relation to the role[s] of TSC22D4 per se and in lipid metabolism of cancer cachexia.

That said, in this paper data presentation is onerously long and could be reduced substantially to make a more clear and focused presentation.

Several results are less highly related [Fig 1E] and could be deleted. Some results are barely or not at all discussed; [Fig 1 E; studies on methionine-choline deficient diet]. Not statistically significant [ie Fig 1D] results could be mentioned but the figures not shown. Some figuers could be reformatted [Fig 1 B,D have huge wide bars with a huge wide space between them; this could be narrowed and made more compact].

Tumor burden is not mentioned at any place in the paper; please add specific tumor mass measurements for animal studies.

Pair feeding was not done - how do we know the lowered VLDL isn't just an effect of lowered food intake?? Pair feeding studies are essential to establish in what degree the specified changes are tumor induced.

With respect to methionine-choline deficient diet; is it possible that the tumor bearing state generates deficiency? See Sengeløv H et al. Inter-relationships between single carbon units' metabolism and resting energy expenditure in weight-losing patients with small cell lung cancer. Effects of methionine supply and chemotherapy. Eur J Cancer. 1994;30A(11):1616-20. Discuss the possibility that this could be happening.

Hard to relate these animal model findings to clinical evidence as there is barely any, but may want to cite Murphy RA, et al. Loss of adipose tissue and plasma phospholipids: relationship to survival in advanced cancer patients. Clin Nutr. 2010 Aug;29(4):482-7.

1st Revision - authors' response 12 October 2012

We thank the referees for their positive and thoughtful comments.
Please find our point-by-point response below.

***** Reviewer's comments *****

Referee #1 (Remarks):
It's an excellent work carried out by a highly qualified group of scientists. Although it is an exclusively experimental study it could be potentially translated, albeit not in the short term, into new therapeutic approaches for the treatment of cancer cachexia in the clinical practice.

We thank the referee for the support.

Referee #2 (Comments on Novelty/Model System):

the lack of pair feeding studies make it unclear as to whether the alterations described are "tumor - induced" at all.

We thank the referee for this important comment.
We agree with the referee that anorexia, i.e. impairment in food intake, is an important feature of the cancer cachectic phenotype. Thus, in order to investigate the metabolic impact of tumor growth independently of anorexia, we have terminated the experiments after reaching a certain degree of cachexia in the animals (roughly 10 % body weight loss). At this relatively early stage, food intake did not differ between the control and tumor-bearing groups as now shown in a new Figure S1B, thereby per se representing a condition of "pair-feeding". Despite no differences in food intake, the animals already showed a clear loss of lean and adipose tissue mass which is due to a tumor-induced overall catabolic metabolic state.
As food intake did not differ between groups any changes in circulating TG levels are unlikely to result from alterations in energy intake.
Furthermore, the VLDL secretion test is routinely done under fasting conditions, thereby demonstrating a clear difference in hepatic lipid handling under food-deprived conditions which can lead to hypotriglyceridemia as reported in this study.
Finally, supernatants of cachexia-inducing tumor cells were found to affect lipogenic gene expression of primary hepatocytes in an isolated co-culture system (Figure 8), demonstrating the capacity of isolated tumors to affect hepatic lipid handling in the absence of ("confounding") systemic food-derived signals.

Referee #2 (Remarks):

Jones et al.
Introduction a broad ramble around various topics; I suggest that this section be completely rewritten with a specific emphasis on the topic of hepatic [lipid] metabolism in cancer cachexia, and culminationg in a statement of hypothesis of the work.

We have shortened the introductory part accordingly and included a statement of hypothesis at the end of this paragraph.

Authors clearly demonstrate a deficit in VLDL formation in murine cancer [colon26] cachexia as well as establishing TSC22D4 as a key regulatory point in hepatic lipid metabolism
Completely new findings on relation to the role[s] of TSC22D4 per se and in lipid metabolism of cancer cachexia.

That said, in this paper data presentation is onerously long and could be reduced substantially to make a more clear and focused presentation.

We have shortened the text accordingly and removed a number of figures from the main manuscript.

Several results are less highly related [Fig 1E] and could be deleted. Some results are barely or not at all discussed; [Fig 1 E; studies on methionine-choline deficient diet]. Not statistically significant [ie Fig 1D] results could be mentioned but the figures not shown. Some figuers could be reformatted [Fig 1 B,D have huge wide bars with a huge wide space between them; this could be narrowed and made more compact].

We have removed Fig. 1D and E as requested. Relevant figures have been re-formatted.

Tumor burden is not mentioned at any place in the paper; please add specific tumor mass measurements for animal studies.

We have included tumor mass measurements as requested (New Figure S1A).

Pair feeding was not done - how do we know the lowered VLDL isn't just an effect of lowered food intake?? Pair feeding studies are essential to establish in what degree the specified changes are tumor induced.

Please see our comment above.

With respect to methionine-choline deficient diet; is it possible that the tumor bearing state generates deficiency? See Sengeløv H et al. Inter-relationships between single carbon units' metabolism and resting energy expenditure in weight-losing patients with small cell lung cancer. Effects of methionine supply and chemotherapy. Eur J Cancer. 1994;30A(11):1616-20. Discuss the possibility that this could be happening.

We thank the referee for this interesting comment. The "deficiency possibility" is now discussed as suggested.

Hard to relate these animal model findings to clinical evidence as there is barely any, but may want to cite Murphy RA, et al. Loss of adipose tissue and plasma phospholipids: relationship to survival in advanced cancer patients. Clin Nutr. 2010 Aug;29(4):482-7.

We have cited the mentioned paper as suggested.

2nd Editorial Decision 22 October 2012

Thank you for the submission of your revised manuscript to EMBO Molecular Medicine. We have now received the enclosed report from the referee who was asked to re-assess it in light of your previous submission.

As you will see, the reviewer is now supportive of publication and I am glad to let you know that we can proceed with the official acceptance of the manuscript after the following editorial points have been addressed:

- The description of all reported data that includes statistical testing must state the name of the statistical test used to generate error bars and P values, the number (n) of independent experiments underlying each data point (not replicate measures of one sample), and the actual P value for each test (not merely 'significant' or 'P < 0.05'). Please see http://onlinelibrary.wiley.com/journal/10.1002/(ISSN)1757-4684/homepage/ForAuthors.html#data2 for more information.

- For experiments involving human subjects the submission must include a statement that informed consent was obtained from all subjects and that the experiments conformed to the principles set out in the WMA Declaration of Helsinki [http://www.wma.net/en/30publications/10policies/b3/] and the NIH Belmont Report [http://ohsr.od.nih.gov/guidelines/belmont.html]. Please see our Guide to Authors for further information and provide the necessary information in the respective Material and Methods part.

Please submit your revised manuscript within two weeks. I look forward to seeing a revised form of your manuscript as soon as possible.

Yours sincerely,

Editor
EMBO Molecular Medicine

***** Reviewer's comments *****

Referee #2 (Comments on Novelty/Model System):

Please refer to previous review.

Referee #2 (Remarks):

The authors have fully corrected the manuscript in function of reviewer comments.

2nd Revision - authors' response 05 November 2012

As requested, we have now additionally stated the names of the statistical tests used to generate error bars and P values, the number (n) of independent experiments, and the actual P value for each test. Corresponding information have been inserted into the figure legends, the individual figure panels, and the materials and methods section.
