# Supplementary material for: TSC22D4 is a molecular output of hepatic wasting metabolism
Source: EMBO Mol Med. 2013 Jan 11;5(2):294–308. doi: 10.1002/emmm.201201869 (PMC3569644; doi:10.1002/emmm.201201869)
Supplement: Supplementary file 2 [file emmm0005-0294-SD2.pdf]

Supplementary Data

Table of contents

Supplementary Table 1

Supplementary Figure Legends

Supplementary Figures S1 – S6

Table 1:

**Fatty acid biosynthesis**	**p= 0,041**
Acacb	
Acaca	
Fasn	
Oxsm	

**Biosynthesis of unsaturated fatty acids**	**p= 0,002**
Pecr	
Acox1	
Elovl6	
Acot2	
Scd1	
Scd2	
Acaa1b	
Acot1	
Elovl2	
Hsd17b12	
Elovl5	
Acot7	
Ptplb	

SUPPLEMENTARY FIGURE LEGENDS

Supplementary figure 1.

a, Tumor mass of Balb/C mice injected with PBS or 1.5x 10^6 colon 26 (C26) cells after 3 weeks (means ± SEM, n≥ 6). b, Daily food intake per mouse in the same mice as in a. (Two Way Repeated Measures ANOVA; Holm-Sidak post hoc) c, Liver cholesterol levels in the same mice as in a. d, Liver triglyceride levels in the same mice as in a. Statistical test c-d: student's t-test.

Supplementary figure 2.

a, Quantitative PCR analysis of hepatic Tsc22d4 RNA levels in mice treated with either PBS or 1.5x 10^6 Colon (C) 26 carcinoma cells (means ± SEM, n≥ 6). b, Body weight of C57Bl/6 mice fed a methionine and choline deficient diet (MCD) or a corresponding control diet for 4 weeks (means ± SEM, n≥ 5, Two Way Repeated Measures ANOVA; Holm-Sidak post hoc). c, Total body fat content of the same mice as in b (means ± SEM, n≥ 5, Two Way Repeated Measures ANOVA; Holm-Sidak method). d, Liver triglyceride levels of the same mice as in b. e, Serum alanine transaminase (ALT) levels in the same mice as in b. f, Blood glucose levels in the same mice as in b. g, Liver weight in the same mice as in b. h, Quantitative PCR analysis of hepatic Tsc22d4 RNA levels in mice fed a low fat diet (LFD) or a high fat diet (HFD) for 11 weeks (means ± SEM, n ≥ 4). Statistical test a, d-h: student's t-test.

Supplementary figure 3.

a-j, Body weight (a), liver weight (b), fat weight (c), lean weight (d), fasting liver triglycerides (e), lipoprotein-associated serum cholesterol (f), liver cholesterol (g), serum non-esterified free fatty acids (NEFA) (h), liver NEFA (i) and serum insulin (j) levels, in control or TSC22D4 shRNA adenovirus–injected C57Bl6 mice 7 days after injection (means ± SEM, n≥7). k-l, Hepatic TSC22D4 RNA (k) and protein (l) levels in TSC22D4 miRNA adeno-associated virus-injected C57Bl6 mice (means ± SEM, n≥5). m-n, Serum triglyceride (m) and cholesterol (n) levels in the same mice as in k. Statistical test a-d, g-k, m-n: student's t-test.

Supplementary figure 4.

a-f, body weight (a), fat weight (b), lean weight (c), liver weight (d), serum cholesterol (e) and liver cholesterol (f) levels in fasted or fed control or TSC22D4 cDNA adenovirus–injected C57Bl6 mice 7 days after injection (means ± SEM, n≥4). g-l, Hepatic TSC22D4 protein levels (g), body weight (h), fat weight (i), lean weight (j), serum cholesterol (k) and liver cholesterol (l) levels in control or TSC22D4 cDNA adenovirus–injected C57Bl6 mice fed either a low fat diet (LFD) or a high fat diet (HFD) for 11 weeks 7 days after injection (means ± SEM, n≥3). Statistical test a-f, h-l: student's t-test.

Supplementary figure 5.

a, Quantitative PCR analysis of lipolysis stimulated lipoprotein receptor (Lsr), low-density lipoprotein receptor (Ldlr) and lipoprotein receptor-related protein (Lrp) mRNA levels in livers of control or TSC22D4 shRNA-injected wild-type C57Bl6 mice (means ± SEM, n≥ 7). b, Quantitative PCR analysis of src-homology protein 1 (Shp1), cholesterol 7 alpha-

hydroxylase (Cyp7a1) in the same mice as in a. c, Quantitative PCR analysis of apolipoprotein C 1 (Apoc1), apolipoprotein C 2 (Apoc2), apolipoprotein A 1 (Apoa1), angiopoietin-like 3 (Angptl3), angiopoietin-like 4 (Angptl4) mRNA levels in the same mice as in a. d, Transient transfection assay of Hepa1c1 hepatocytes cotransfected with a pGL2_FAS -150bp/-43bp FasN promoter construct and a construct expressing TSC22D4 cDNA (means ± SEM, $n \geq 3$, Two Way ANOVA; Holm-Sidak post hoc). e, Western blot of liver, extracts from the same mice as in a using Lipin1, Acly and VCP antibodies. Statistical test a-c: student's t-test.

Supplementary figure 6.

a, Correlation of Peroxisome proliferator-activated receptor gamma coactivator 1-alpha (PGC1a) and Transducin beta like 1 (TBL1) RNA expression and weight loss in mice treated with either PBS or 1.5x 10^6 Colon (C) 26 carcinoma cells (means ± SEM, $n \geq 6$). b, Nuclear receptor coactivator 1 (Src1), Peroxisome proliferator-activated receptor gamma coactivator 1-beta (PGC1b), Creb binding protein (CBP) and nuclear receptor coactivator 3 (Src3), RNA expression weight loss in the same mice as in a. Statistical test a-b: Pearson correlation coefficient, F-test to determine significance.

Figure S1

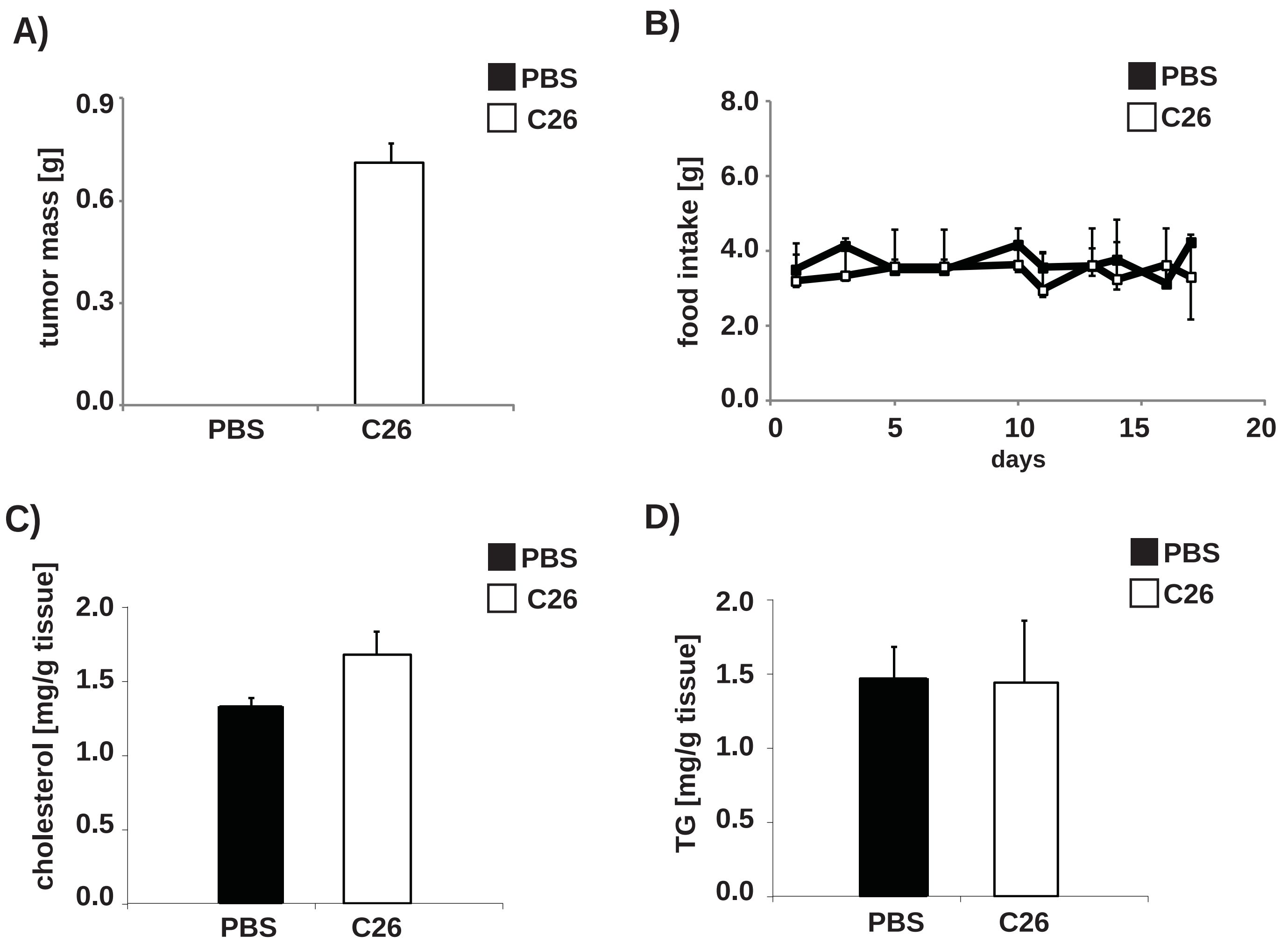
A)
PBS
C26
tumor mass [g]
0.9
0.6
0.3
0.0
PBS
C26
B)
PBS
C26
food intake [g]
8.0
6.0
4.0
2.0
0.0
0
5
10
15
20
days
C)
PBS
C26
cholesterol [mg/g tissue]
2.0
1.5
1.0
0.5
0.0
PBS
C26
D)
PBS
C26
TG [mg/g tissue]
2.0
1.5
1.0
0.5
0.0
PBS
C26

Figure S2

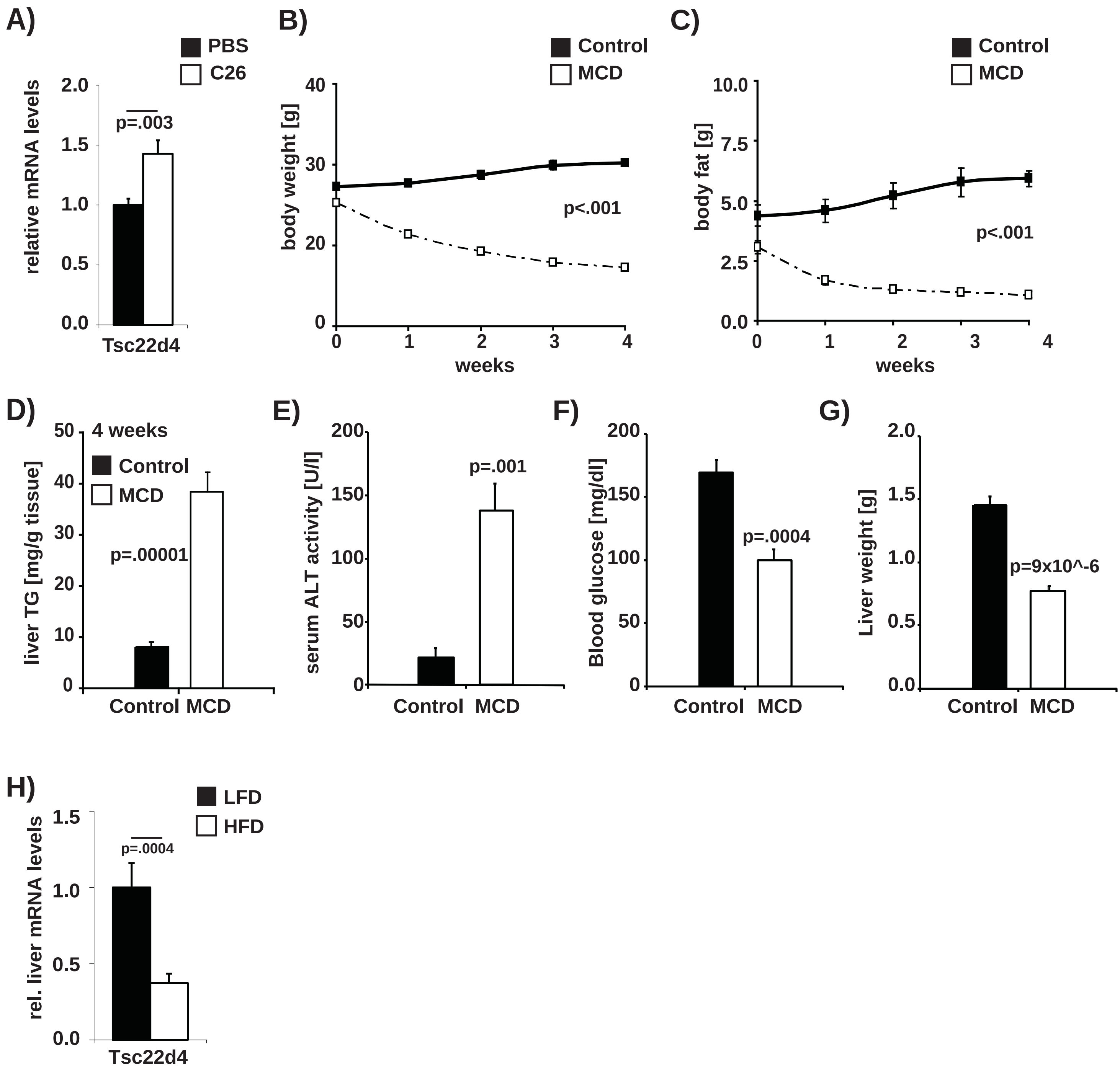

A)
PBS
C26
relative mRNA levels
p=.003
Tsc22d4
B)
Control
MCD
body weight [g]
p<.001
weeks
C)
Control
MCD
body fat [g]
p<.001
weeks
D)
4 weeks
Control
MCD
liver TG [mg/g tissue]
p=.00001
Control MCD
E)
serum ALT activity [U/l]
p=.001
Control MCD
F)
Blood glucose [mg/dl]
p=.0004
Control MCD
G)
Liver weight [g]
p=9x10^-6
Control MCD
H)
LFD
HFD
rel. liver mRNA levels
p=.0004
Tsc22d4

Figure S3

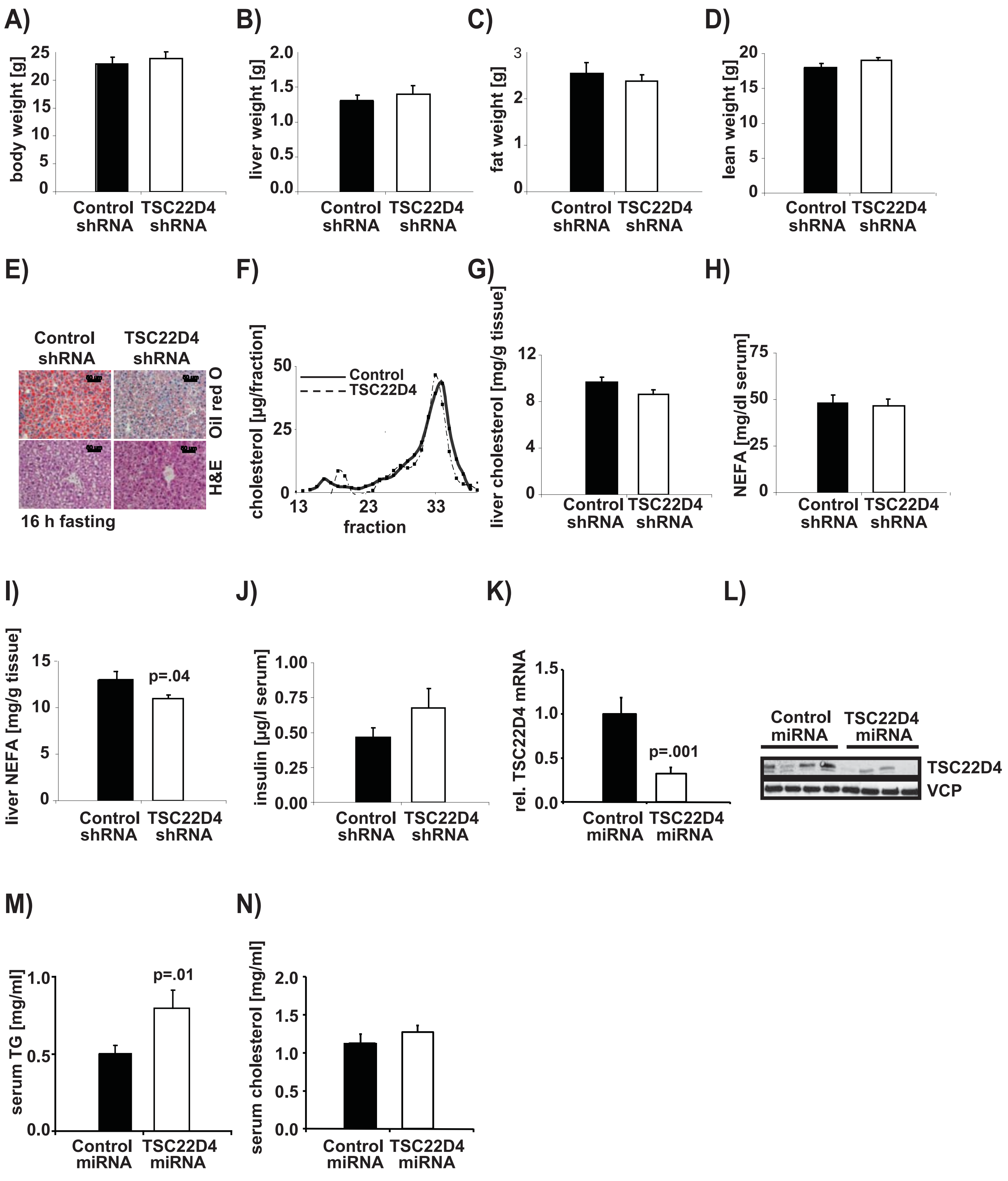
A)
body weight [g]
Control shRNA
TSC22D4 shRNA
B)
liver weight [g]
C)
fat weight [g]
D)
lean weight [g]
E)
Control shRNA
TSC22D4 shRNA
Oil red O
H&E
16 h fasting
F)
cholesterol [µg/fraction]
Control
TSC22D4
fraction
G)
liver cholesterol [mg/g tissue]
H)
NEFA [mg/dl serum]
I)
liver NEFA [mg/g tissue]
p=.04
J)
insulin [µg/l serum]
K)
rel. TSC22D4 mRNA
p=.001
Control miRNA
TSC22D4 miRNA
L)
TSC22D4
VCP
M)
serum TG [mg/ml]
p=.01
N)
serum cholesterol [mg/ml]

Figure S4

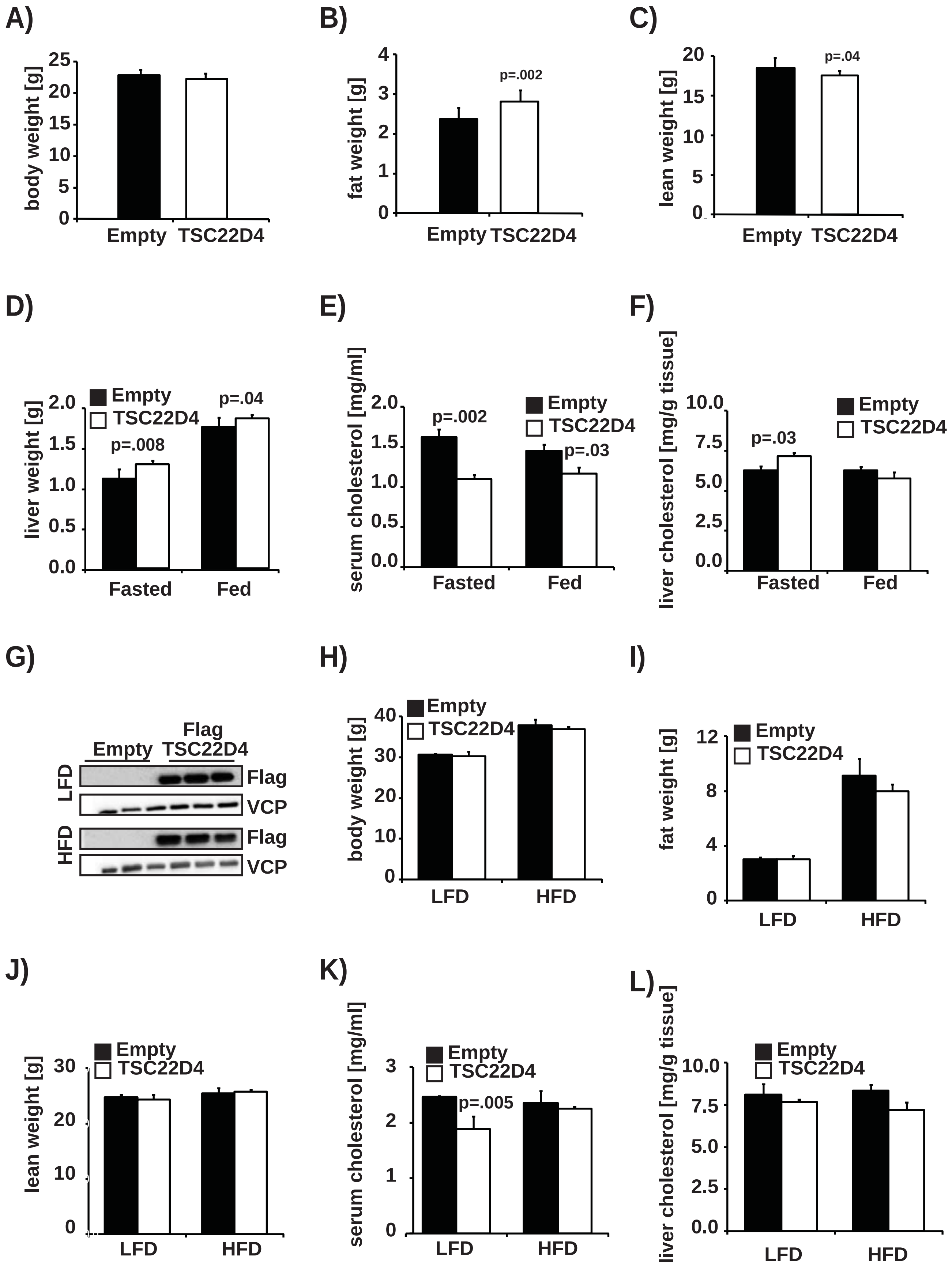

A)
body weight [g]
25
20
15
10
5
0
Empty
TSC22D4
B)
fat weight [g]
p=.002
4
3
2
1
0
Empty
TSC22D4
C)
lean weight [g]
p=.04
20
15
10
5
0
Empty
TSC22D4
D)
liver weight [g]
Empty
TSC22D4
p=.008
p=.04
2.0
1.5
1.0
0.5
0.0
Fasted
Fed
E)
serum cholesterol [mg/ml]
Empty
TSC22D4
p=.002
p=.03
2.0
1.5
1.0
0.5
0.0
Fasted
Fed
F)
liver cholesterol [mg/g tissue]
Empty
TSC22D4
p=.03
10.0
7.5
5.0
2.5
0.0
Fasted
Fed
G)
Empty
Flag TSC22D4
LFD
HFD
Flag
VCP
Flag
VCP
H)
body weight [g]
Empty
TSC22D4
40
30
20
10
0
LFD
HFD
I)
fat weight [g]
Empty
TSC22D4
12
8
4
0
LFD
HFD
J)
lean weight [g]
Empty
TSC22D4
30
20
10
0
LFD
HFD
K)
serum cholesterol [mg/ml]
Empty
TSC22D4
p=.005
3
2
1
0
LFD
HFD
L)
liver cholesterol [mg/g tissue]
Empty
TSC22D4
10.0
7.5
5.0
2.5
0.0
LFD
HFD

Figure S5

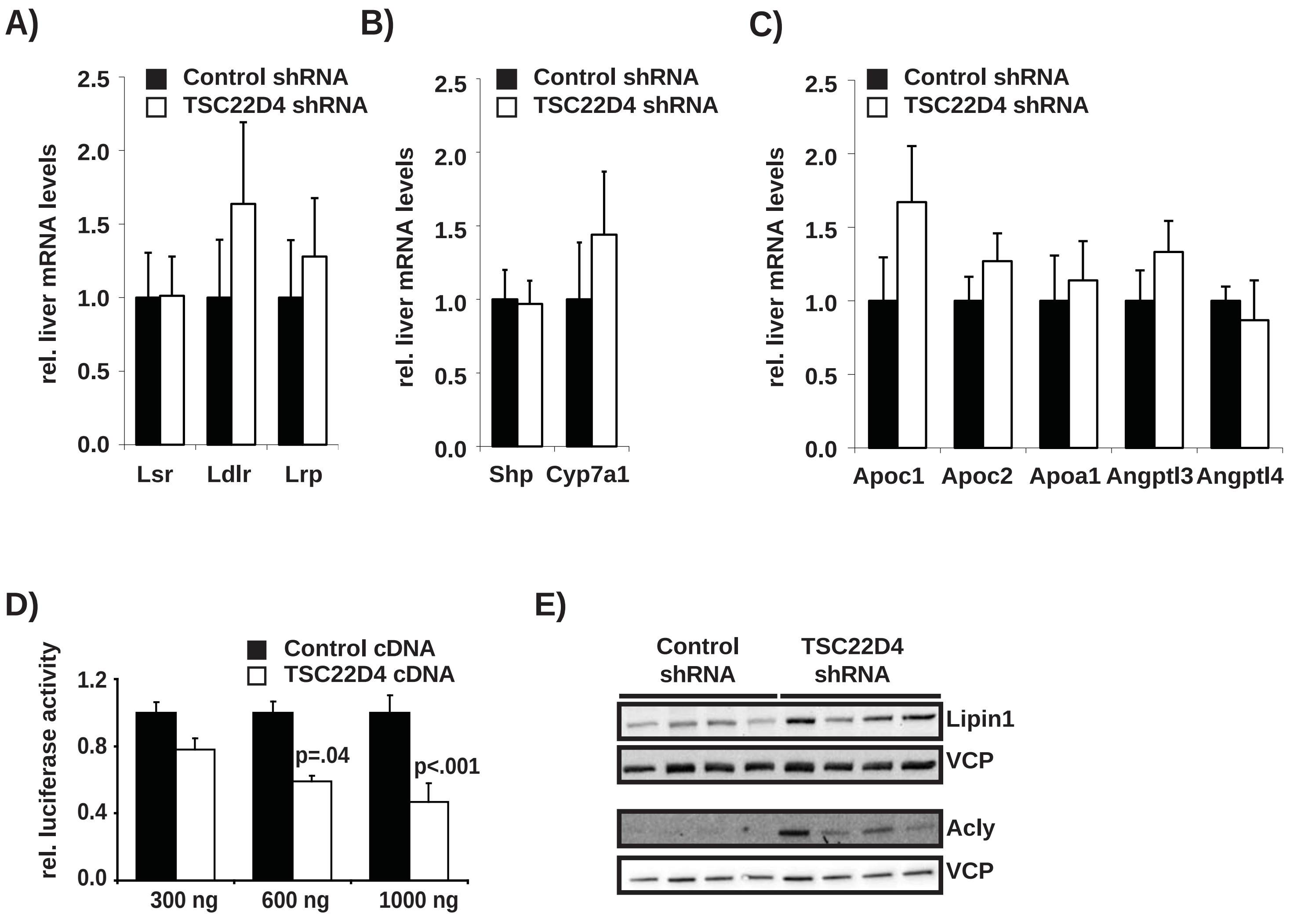

A)
B)
C)
Control shRNA
TSC22D4 shRNA
rel. liver mRNA levels
2.5
2.0
1.5
1.0
0.5
0.0
Lsr
Ldlr
Lrp
Shp
Cyp7a1
Apoc1
Apoc2
Apoa1
Angptl3
Angptl4
D)
Control cDNA
TSC22D4 cDNA
rel. luciferase activity
1.2
0.8
0.4
0.0
p=.04
p<.001
300 ng
600 ng
1000 ng
E)
Control shRNA
TSC22D4 shRNA
Lipin1
VCP
Acly
VCP

Figure S6

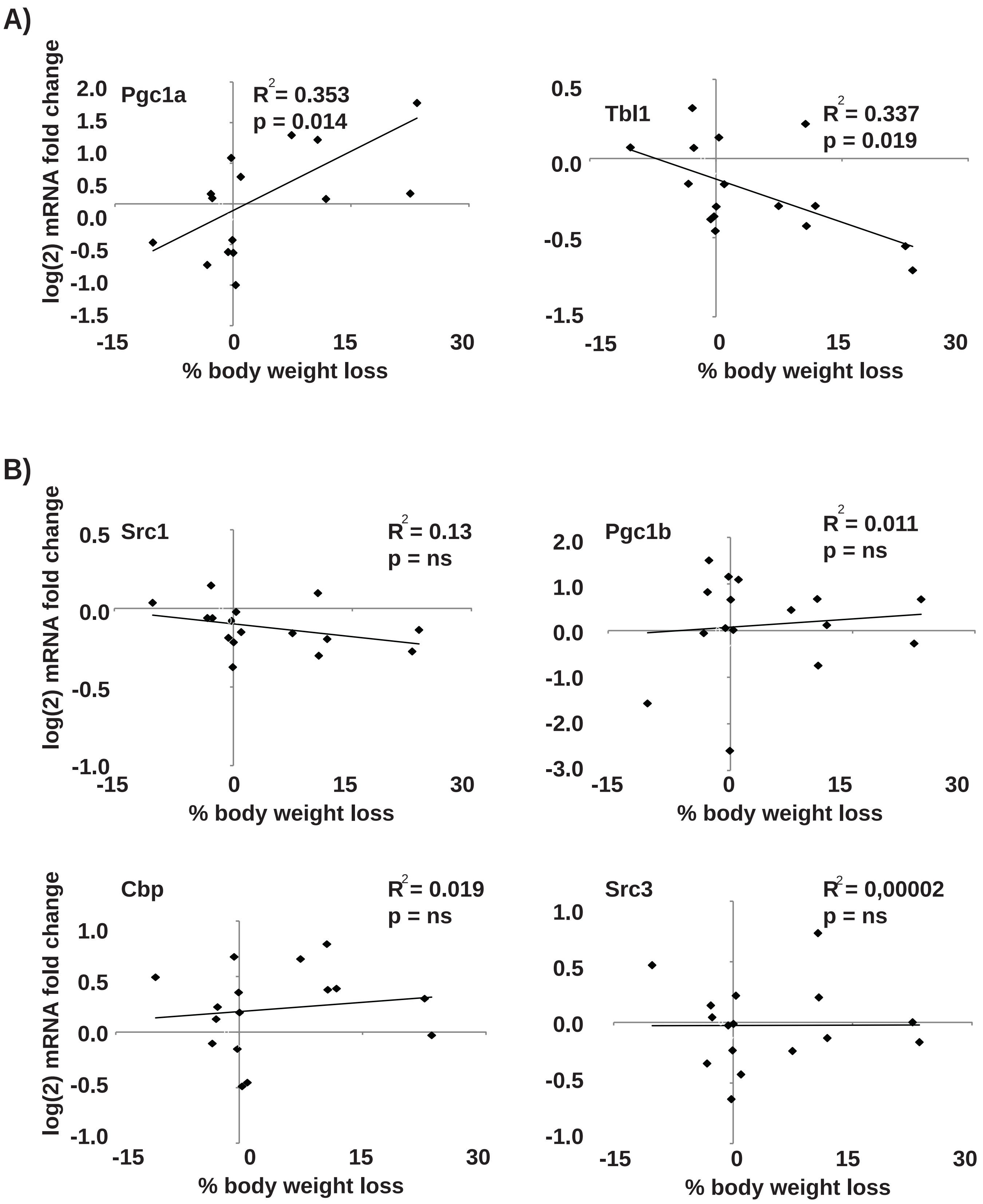
A)
Pgc1a
R2 = 0.353
p = 0.014
Tbl1
R2 = 0.337
p = 0.019
log(2) mRNA fold change
% body weight loss
-15
0
15
30
2.0
1.5
1.0
0.5
0.0
-0.5
-1.0
-1.5
B)
Src1
R2 = 0.13
p = ns
Pgc1b
R2 = 0.011
p = ns
Cbp
R2 = 0.019
p = ns
Src3
R2 = 0,00002
p = ns
-2.0
-3.0
